# Supplementary material for: Acceptability of self-sampling human papillomavirus test for cervical cancer screening in Japan: A questionnaire survey in the ACCESS trial
Source: PLoS One. 2023 Jun 8;18(6):e0286909. doi: 10.1371/journal.pone.0286909 (PMC10249862; doi:10.1371/journal.pone.0286909)
Supplement: S1 File — (DOCX) [file pone.0286909.s001.docx]

# Supplementary Tables

**Title:** Acceptability of self-sampling human papillomavirus test for cervical cancer screening in Japan: A questionnaire survey in the ACCESS trial

**Authors:** Misuzu Fujita, Kengo Nagashima, Minobu Shimazu, Misae Suzuki, Ichiro Tauchi, Miwa Sakuma, Setsuko Yamamoto, Hideki Hanaoka, Makio Shozu, Nobuhide Tsuruoka, Tokuzo Kasai, Akira Hata

**Corresponding author**

E-mail: [mi-hujita@kenko-chiba.or.jp](mailto:mi-hujita@kenko-chiba.or.jp) (MF)

# S1 Table

Proportion of participants who agreed with the item “Sample taking was painful”

|  | All ^a^ | N ^b^ | Percent (95% CI) | OR (95% CI) | p-value | p for trend |
| --- | --- | --- | --- | --- | --- | --- |
| Age |  |  |  |  |  |  |
| 30−39 years | 371 | 60 | 16.2 (12.6−20.3) | 1.00 | <0.001 | 0.002 |
| 40−49 years | 428 | 54 | 12.6 (9.6−16.1) | 0.75 (0.50−1.11) |  |  |
| 50−59 years | 340 | 88 | 25.9 (21.3−30.9) | 1.81 (1.25−2.61) |  |  |
| Duration without screening |  |  |  |  |  |  |
| 3−5 years | 195 | 29 | 14.9 (10.2−20.7) | 1.00 | 0.185 | 0.148 |
| 6 years or more | 265 | 41 | 15.5 (11.3−20.4) | 1.05 (0.63−1.76) |  |  |
| No screening records | 679 | 132 | 19.4 (16.5−22.6) | 1.38 (0.89−2.14) |  |  |

CI: confidence interval; OR: odds ratio

^a^ Participants who answered this question.

^b^ Number of participants who answered “Fully agree” or “Somewhat agree”.

# S2 Table

Proportion of participants who agreed with the item “Sample taking was uncomfortable”

|  | All ^a^ | N ^b^ | Percent (95% CI) | OR (95% CI) | p-value | p for trend |
| --- | --- | --- | --- | --- | --- | --- |
| Age |  |  |  |  |  |  |
| 30−39 years | 372 | 49 | 13.2 (9.9−17.0) | 1.00 | 0.519 | 0.655 |
| 40−49 years | 426 | 45 | 10.6 (7.8−13.9) | 0.78 (0.51−1.20) |  |  |
| 50−59 years | 340 | 41 | 12.1 (8.8−16.0) | 0.90 (0.58−1.41) |  |  |
| Duration without screening |  |  |  |  |  |  |
| 3−5 years | 196 | 16 | 8.2 (4.7−12.9) | 1.00 | 0.052 | 0.040 |
| 6 years or more | 265 | 26 | 9.8 (6.5−14.0) | 1.22 (0.64−2.35) |  |  |
| No screening records | 677 | 93 | 13.7 (11.2−16.6) | 1.79 (1.03−3.12) |  |  |

CI: confidence interval; OR: odds ratio

^a^ Participants who answered this question.

^b^ Number of participants who answered “Fully agree” or “Somewhat agree”.

# S3 Table

Proportion of participants who agreed with the item “Sample taking was embarrassing”

|  | All ^a^ | N ^b^ | Percent (95% CI) | OR (95% CI) | p-value | p for trend |
| --- | --- | --- | --- | --- | --- | --- |
| Age |  |  |  |  |  |  |
| 30−39 years | 371 | 13 | 3.5 (1.9−5.9) | 1.00 | 0.465 | 0.379 |
| 40−49 years | 428 | 17 | 4.0 (2.3−6.3) | 1.14 (0.54−2.38) |  |  |
| 50−59 years | 337 | 8 | 2.4 (1.0−4.6) | 0.67 (0.27−1.64) |  |  |
| Duration without screening |  |  |  |  |  |  |
| 3−5 years | 195 | 7 | 3.6 (1.5−7.3) | 1.00 | 0.971 | 0.816 |
| 6 years or more | 264 | 9 | 3.4 (1.6−6.4) | 0.95 (0.35−2.59) |  |  |
| No screening records | 677 | 22 | 3.2 (2.0−4.9) | 0.90 (0.38−2.14) |  |  |

CI: confidence interval; OR: odds ratio

^a^ Participants who answered this question.

^b^ Number of participants who answered “Fully agree” or “Somewhat agree”.

# S4 Table

Proportion of participants who agreed with the item “Sample taking was easy”

|  | All ^a^ | N ^b^ | Percent (95% CI) | OR (95% CI) | p-value | p for trend |
| --- | --- | --- | --- | --- | --- | --- |
| Age |  |  |  |  |  |  |
| 30−39 years | 371 | 303 | 81.7 (77.4−85.5) | 1.00 | 0.025 | 0.030 |
| 40−49 years | 427 | 351 | 82.2 (78.2−85.7) | 1.04 (0.72−1.49) |  |  |
| 50−59 years | 339 | 254 | 74.9 (70.0−79.5) | 0.67 (0.47−0.96) |  |  |
| Duration without screening |  |  |  |  |  |  |
| 3−5 years | 196 | 158 | 80.6 (74.4−85.9) | 1.00 | 0.910 | 0.719 |
| 6 years or more | 265 | 213 | 80.4 (75.1−85.0) | 0.99 (0.62−1.57) |  |  |
| No screening records | 676 | 537 | 79.4 (76.2−82.4) | 0.93 (0.62−1.39) |  |  |

CI: confidence interval; OR: odds ratio

^a^ Participants who answered this question.

^b^ Number of participants who answered “Fully agree” or “Somewhat agree”.

# S5 Table

Proportion of participants who agreed with the item “Sample taking was convenient”

|  | All ^a^ | N ^b^ | Percent (95% CI) | OR (95% CI) | p-value | p for trend |
| --- | --- | --- | --- | --- | --- | --- |
| Age |  |  |  |  |  |  |
| 30−39 years | 369 | 291 | 78.9 (74.3−82.9) | 1.00 | 0.290 | 0.475 |
| 40−49 years | 427 | 347 | 81.3 (77.2−84.9) | 1.16 (0.82−1.65) |  |  |
| 50−59 years | 338 | 259 | 76.6 (71.7−81.0) | 0.88 (0.62−1.25) |  |  |
| Duration without screening |  |  |  |  |  |  |
| 3−5 years | 196 | 160 | 81.6 (75.5−86.8) | 1.00 | 0.619 | 0.328 |
| 6 years or more | 262 | 207 | 79.0 (73.6−83.8) | 0.85 (0.53−1.35) |  |  |
| No screening records | 676 | 530 | 78.4 (75.1−81.4) | 0.82 (0.54−1.22) |  |  |

CI: confidence interval; OR: odds ratio

^a^ Participants who answered this question.

^b^ Number of participants who answered “Fully agree” or “Somewhat agree”.

# S6 Table

Proportion of participants who agreed with the item “The user instructions were clear”

|  | All ^a^ | N ^b^ | Percent (95% CI) | OR (95% CI) | p-value | p for trend |
| --- | --- | --- | --- | --- | --- | --- |
| Age |  |  |  |  |  |  |
| 30−39 years | 372 | 321 | 86.3 (82.4−89.6) | 1.00 | 0.055 | 0.054 |
| 40−49 years | 429 | 372 | 86.7 (83.1−89.8) | 1.04 (0.69−1.56) |  |  |
| 50−59 years | 341 | 276 | 80.9 (76.4−85.0) | 0.67 (0.45−1.01) |  |  |
| Duration without screening |  |  |  |  |  |  |
| 3−5 years | 196 | 166 | 84.7 (78.9−89.4) | 1.00 | 0.985 | 0.916 |
| 6 years or more | 266 | 225 | 84.6 (79.7−88.7) | 0.99 (0.59−1.65) |  |  |
| No screening records | 680 | 578 | 85.0 (82.1−87.6) | 1.02 (0.66−1.59) |  |  |

CI: confidence interval; OR: odds ratio

^a^ Participants who answered this question.

^b^ Number of participants who answered “Fully agree” or “Somewhat agree”.

# S7 Table

Proportion of participants who agreed with the item “The device worked as the instructions explained”

|  | All ^a^ | N ^b^ | Percent (95% CI) | OR (95% CI) | p-value | p for trend |
| --- | --- | --- | --- | --- | --- | --- |
| Age |  |  |  |  |  |  |
| 30−39 years | 371 | 308 | 83.0 (78.8−86.7) | 1.00 | 0.126 | 0.084 |
| 40−49 years | 429 | 356 | 83.0 (79.1−86.4) | 1.00 (0.69−1.44) |  |  |
| 50−59 years | 339 | 264 | 77.9 (73.1−82.2) | 0.72 (0.50−1.05) |  |  |
| Duration without screening |  |  |  |  |  |  |
| 3−5 years | 195 | 156 | 80.0 (73.7−85.4) | 1.00 | 0.680 | 0.463 |
| 6 years or more | 266 | 214 | 80.5 (75.2−85.0) | 1.03 (0.65−1.64) |  |  |
| No screening records | 678 | 558 | 82.3 (79.2−85.1) | 1.16 (0.78−1.74) |  |  |

CI: confidence interval; OR: odds ratio

^a^ Participants who answered this question.

^b^ Number of participants who answered “Fully agree” or “Somewhat agree”.

# S8 Table

Proportion of participants who agreed with the item “I believe I was successful in taking the sample”

|  | All ^a^ | N ^b^ | Percent (95% CI) | OR (95% CI) | p-value | p for trend |
| --- | --- | --- | --- | --- | --- | --- |
| Age |  |  |  |  |  |  |
| 30−39 years | 373 | 83 | 22.3 (18.1−26.8) | 1.00 | 0.709 | 0.685 |
| 40−49 years | 428 | 90 | 21.0 (17.3−25.2) | 0.93 (0.66−1.30) |  |  |
| 50−59 years | 340 | 80 | 23.5 (19.1−28.4) | 1.08 (0.76−1.53) |  |  |
| Duration without screening |  |  |  |  |  |  |
| 3−5 years | 196 | 56 | 28.6 (22.4−35.4) | 1.00 | 0.027 | 0.053 |
| 6 years or more | 265 | 48 | 18.1 (13.7−23.3) | 0.55 (0.36−0.86) |  |  |
| No screening records | 680 | 149 | 21.9 (18.9−25.2) | 0.70 (0.49−1.00) |  |  |

CI: confidence interval; OR: odds ratio

^a^ Participants who answered this question.

^b^ Number of participants who answered “Fully agree” or “Somewhat agree”.

# S9 Table

Sensitivity analysis for willingness to undergo screening per type of sample collection

|  | All | N ^d^ | Percent (95 % CI) | OR (95 % CI) | p-value |
| --- | --- | --- | --- | --- | --- |
| Screening with doctor-collected samples |  |  |  |  |  |
| All | 860 ^a^ | 374 | 43.5 (40.1−46.9) | ― | <0.001 ^e^ |
| Age |  |  |  |  |  |
| 30−39 years | 304 ^b^ | 173 | 56.9 (51.1−62.5) | 1.00 | <0.001 ^f^ |
| 40−49 years | 317 ^b^ | 144 | 45.4 (39.9−51.1) | 0.63 (0.46−0.87) | <0.001 ^g^ |
| 50−59 years | 247 ^b^ | 61 | 24.7 (19.4−30.6) | 0.25 (0.17−0.36) |  |
| Duration without screening |  |  |  |  |  |
| 3−5 years | 162 ^b^ | 92 | 56.8 (48.8−64.5) | 1.00 | 0.001 ^f^ |
| 6 years or more | 193 ^b^ | 84 | 43.5 (36.4−50.8) | 0.59 (0.38−0.89) | <0.001 ^g^ |
| No screening records | 513 ^b^ | 202 | 39.4 (35.1−43.8) | 0.49 (0.35−0.71) |  |
| Screening with self-collected samples |  |  |  |  |  |
| All | 860 ^a^ | 767 | 89.2 (86.9−91.2) | ― |  |
| Age |  |  |  |  |  |
| 30−39 years | 309 ^c^ | 282 | 91.3 (87.5−94.2) | 1.00 | 0.292 ^f^ |
| 40−49 years | 327 ^c^ | 288 | 88.1 (84.1−91.4) | 0.71 (0.42−1.19) | 0.152 ^g^ |
| 50−59 years | 257 ^c^ | 225 | 87.5 (82.9−91.3) | 0.67 (0.39−1.16) |  |
| Duration without screening |  |  |  |  |  |
| 3−5 years | 165 ^c^ | 147 | 89.1 (83.3−93.4) | 1.00 | 0.555 ^f^ |
| 6 years or more | 201 ^c^ | 183 | 91.0 (86.2−94.6) | 1.24 (0.63−2.48) | 0.764 ^g^ |
| No screening records | 527 ^c^ | 465 | 88.2 (85.2−90.9) | 0.92 (0.53−1.60) |  |

CI: confidence interval; OR: odds ratio. Participants who selected “I had an opportunity to undergo cytology testing other than the screening provided by the city” as the reason for not undergoing screening were excluded.

Remaining participants were 911.

^a^ Number of participants who answered both questions related to willingness to undergo screening with doctor-collected samples and self-collected samples. Missing values were 51.

^b^ Number of participants who answered a question related to willingness to undergo screening with doctor-collected samples. Missing values were 43.

^c^ Number of participants who answered a question related to willingness to undergo screening with self-collected samples. Missing values were 18.

^d^ Number of participants who answered “I will receive.”

^e^ Comparison of willingness between two screenings using McNemer’s test.

^f^ Comparison of willingness with age or duration without screening using chi-squared test.

^g^ Comparison of willingness with age or duration without screening using linear trend test.
