# Supplementary material for: Acceptability of self-sampling human papillomavirus test for cervical cancer screening in Japan: A questionnaire survey in the ACCESS trial
Source: PLoS One. 2023 Jun 8;18(6):e0286909. doi: 10.1371/journal.pone.0286909 (PMC10249862; doi:10.1371/journal.pone.0286909)
Supplement: S1 Checklist — (DOCX) [file pone.0286909.s003.docx]

STROBE Statement—checklist of items that should be included in reports of observational studies

|  | Item No. | Recommendation | Page  No. | Relevant text from manuscript |
| --- | --- | --- | --- | --- |
| **Title and abstract** | 1 | (*a*) Indicate the study’s design with a commonly used term in the title or the abstract | 1 | Questionnaire survey in the ACCESS trial |
|  |  | (*b*) Provide in the abstract an informative and balanced summary of what was done and what was found | 3, 4 | This study, as a secondary analysis of the trial, explores the acceptability and preference of self-sampling.  High acceptability of the self-sampling HPV test was confirmed, while concerns about self-sampling procedures remained. Screening with a self-collected sample was preferred than that with a doctor-collected sample and the former might alleviate disparities in screening rates. |
| Introduction | | | |  |
| Background/rationale | 2 | Explain the scientific background and rationale for the investigation being reported | 4, 5 | See the Introduction section. |
| Objectives | 3 | State specific objectives, including any prespecified hypotheses | 5 | We conducted a pre-planned questionnaire survey as a part of this trial to examine the main reasons for not undergoing cervical cancer screening, knowledge about HPV, acceptability of the self-sampling HPV test, and screening preference in future in Japanese women. |
| Methods | | | |  |
| Study design | 4 | Present key elements of study design early in the paper | 6 | This study is a questionnaire survey linked with the ACCESS trial, an ongoing randomized controlled trial. |
| Setting | 5 | Describe the setting, locations, and relevant dates, including periods of recruitment, exposure, follow-up, and data collection | 6, 7 | See the Participants section. |
| Participants | 6 | (*a*) *Cohort study*—Give the eligibility criteria, and the sources and methods of selection of participants. Describe methods of follow-up  *Case-control study*—Give the eligibility criteria, and the sources and methods of case ascertainment and control selection. Give the rationale for the choice of cases and controls  *Cross-sectional study*—Give the eligibility criteria, and the sources and methods of selection of participants | 6, 7 | Inclusion criteria were 1) Women living in Ichihara City as of December 22, 2020; 2) women aged 30−59 years as of April 1, 2021; 3) women who were the target population for cervical cancer screening by Ichihara City in 2021; and 4) women who had not received routine cervical cancer screening provided by Ichihara City for three years or more.  The participants of this study were those who submitted all the following three items by September 3, 2021: filled consent form, self-collected sample, and filled questionnaire. |
|  |  | (*b*) *Cohort study*—For matched studies, give matching criteria and number of exposed and unexposed  *Case-control study*—For matched studies, give matching criteria and the number of controls per case | N/A |  |
| Variables | 7 | Clearly define all outcomes, exposures, predictors, potential confounders, and effect modifiers. Give diagnostic criteria, if applicable | 7, 8 | See the Questionnaire and Other data sections. |
| Data sources/ measurement | 8* | For each variable of interest, give sources of data and details of methods of assessment (measurement). Describe comparability of assessment methods if there is more than one group | N/A |  |
| Bias | 9 | Describe any efforts to address potential sources of bias | N/A |  |
| Study size | 10 | Explain how the study size was arrived at | 6 | The sample size was determined based on the primary endpoint in the ACCESS trial. |

Continued on next page

| Quantitative variables | 11 | Explain how quantitative variables were handled in the analyses. If applicable, describe which groupings were chosen and why | 8, 9 | See the Statistical analysis section. |
| --- | --- | --- | --- | --- |
| Statistical methods | 12 | (*a*) Describe all statistical methods, including those used to control for confounding | 8, 9 | See the Statistical analysis section. |
|  |  | (*b*) Describe any methods used to examine subgroups and interactions | N/A |  |
|  |  | (*c*) Explain how missing data were addressed | 9 | Missing values were included in the aggregate but excluded in the statistical test. |
|  |  | (*d*) *Cohort study*—If applicable, explain how loss to follow-up was addressed  *Case-control study*—If applicable, explain how matching of cases and controls was addressed  *Cross-sectional study*—If applicable, describe analytical methods taking account of sampling strategy | N/A |  |
|  |  | (*e*) Describe any sensitivity analyses | 9 | As a sensitivity analysis, the willingness was compared excluding participants who selected “I had an opportunity to undergo cytology testing other than the screening provided by the city” as the reason for not undergoing screening. |
| Results | | | | |
| Participants | 13* | (a) Report numbers of individuals at each stage of study—eg numbers potentially eligible, examined for eligibility, confirmed eligible, included in the study, completing follow-up, and analysed | 10 | Of the 7,340 participants in the self-sampling arm, 1,372 (18.7%) ordered the self-sampling HPV test, and 1,196 (16.3%) returned both the filled consent form and a self-collected sample. Of those, 1,192 (99.7%) also returned the filled questionnaire as shown in Fig 1. |
|  |  | (b) Give reasons for non-participation at each stage | 10 | Of the 7,340 participants in the self-sampling arm, 1,372 (18.7%) ordered the self-sampling HPV test, and 1,196 (16.3%) returned both the filled consent form and a self-collected sample. Of those, 1,192 (99.7%) also returned the filled questionnaire as shown in Fig 1. |
|  |  | (c) Consider use of a flow diagram | 10 | Fig 1. |
| Descriptive data | 14* | (a) Give characteristics of study participants (eg demographic, clinical, social) and information on exposures and potential confounders | 10 | Characteristics of the participants are shown in Table 1. Mean (standard deviation) age was 44.1 (8.2) years. Majority of the participants (59.6%) had no registration of receiving cervical cancer screening in the database of Ichihara City Hall. |
|  |  | (b) Indicate number of participants with missing data for each variable of interest | 11-16 | Table 2 and 3 |
|  |  | (c) *Cohort study*—Summarise follow-up time (eg, average and total amount) | N/A |  |
| Outcome data | 15* | *Cohort study*—Report numbers of outcome events or summary measures over time | N/A |  |
|  |  | *Case-control study—*Report numbers in each exposure category, or summary measures of exposure | N/A |  |
|  |  | *Cross-sectional study—*Report numbers of outcome events or summary measures | 11-16 | Table 2 and 3 |
| Main results | 16 | (*a*) Give unadjusted estimates and, if applicable, confounder-adjusted estimates and their precision (eg, 95% confidence interval). Make clear which confounders were adjusted for and why they were included | N/A |  |
|  |  | (*b*) Report category boundaries when continuous variables were categorized | 11-16 | Table 2 and 3 |
|  |  | (*c*) If relevant, consider translating estimates of relative risk into absolute risk for a meaningful time period | N/A |  |

Continued on next page

| Other analyses | 17 | Report other analyses done—eg analyses of subgroups and interactions, and sensitivity analyses | N/A |  |
| --- | --- | --- | --- | --- |
| Discussion | | | | |
| Key results | 18 | Summarise key results with reference to study objectives | 21 | In conclusion, this study reveals high acceptability and preference of the self-sampling HPV test for cervical cancer screening in future and adds further evidence to strengthen a hypothesis that the test is acceptable to women across cultures and countries. Additionally, this study suggests that the self-sampling HPV test reduces disparities in knowledge about HPV and screening rates. |
| Limitations | 19 | Discuss limitations of the study, taking into account sources of potential bias or imprecision. Discuss both direction and magnitude of any potential bias | 20, 21 | See the second paragraph from the bottom in the Discussion section. |
| Interpretation | 20 | Give a cautious overall interpretation of results considering objectives, limitations, multiplicity of analyses, results from similar studies, and other relevant evidence | 17-21 | See the discussion section. |
| Generalisability | 21 | Discuss the generalisability (external validity) of the study results | 20, 21 | See the second paragraph from the bottom in the Discussion section. |
| Other information | |  | | |
| Funding | 22 | Give the source of funding and the role of the funders for the present study and, if applicable, for the original study on which the present article is based | In the submission system | This work was supported by a Japan Society for the Promotion of Science (JSPS) KAKENHI Grant (AH, Number 20H03906, <http://www.jsps.go.jp/j-grantsinaid>). |

*Give information separately for cases and controls in case-control studies and, if applicable, for exposed and unexposed groups in cohort and cross-sectional studies.

**Note:** An Explanation and Elaboration article discusses each checklist item and gives methodological background and published examples of transparent reporting. The STROBE checklist is best used in conjunction with this article (freely available on the Web sites of PLoS Medicine at http://www.plosmedicine.org/, Annals of Internal Medicine at http://www.annals.org/, and Epidemiology at http://www.epidem.com/). Information on the STROBE Initiative is available at www.strobe-statement.org.
