# Supplementary material for: Acceptability of self-sampling human papillomavirus test for cervical cancer screening in Japan: A questionnaire survey in the ACCESS trial
Source: PLoS One. 2023 Jun 8;18(6):e0286909. doi: 10.1371/journal.pone.0286909 (PMC10249862; doi:10.1371/journal.pone.0286909)
Supplement: Attachment — Submitted filename: Response_to_Reviewers_20230330.docx [file pone.0286909.s004.docx]

# Responses to the Editor and Reviewers’ Comments

Submission ID: PONE-D-22-28147

Title: Acceptability of self-sampling human papillomavirus test for cervical cancer screening in Japan: A questionnaire survey in the ACCESS trial

We appreciate the Editor’s and Reviewers’ comments toward improving our paper. We have revised the manuscript to address all the comments, and our point-by-point responses are provided below. We have included the **page and line numbers of the revisions made in the main text**. Revisions in response to the Editor’s and Reviewers’ comments are **highlighted in yellow** in the revised manuscript with track changes.

# Point-by-point responses

# Academic editor

# Comment 1

English writing needs to be improved. Awkward or ambiguous English expressions and incorrect grammar are seen in current version, e.g. the 3rd sentence in Methods of Abstract, the last sentence in Results of Abstract, ... The authors are advised please to check thoroughly the entire manuscript for language improvement, and/or ask help for English editing if available at your own end.

# Response 1

Thank you for your comment. Although we had asked an English editing company to edit our English, some incorrect expressions remained. We checked the entire manuscript and asked the company to re-edit our manuscript.

# Comment 2

The current manuscript is unnecessarily lengthy. Please check the reviewers’ comments as well. Please write concisely in the revision with removing irrelevant sentences/paragraphs and duplicated/redundant materials.

# Response 2

In line with comments from reviewers, we changed Table 2 to a graph (from page 12, lines 214–215) and deleted the descriptions based on individual responses in the Results section and those related to COVID-19 in the Discussion section. Additionally, some sentences in the Discussion section were deleted.

# Comment 3

Although the parenting study is an ongoing control randomized trial, however the current manuscript analyzed a subset of the data from one arm only. Please make this crystal clear, specially in abstract and here there when it needs to be clarified.

# Response 3

We added an explanation in the Abstract (page 3, lines 31–32) and revised the description in the Materials and Methods section (page 7, lines 126–129).

# Comment 4

Please look at Reviewer 2’s comment about the sampling bias concern. Please define your study population explicitly and give the limitation where your findings can be generalized to.

# Response 4

Thank you for your comment. In line with a comment from Reviewer 2, we revised Table 1 (from page 10, line 199, to page 11, line 204) and added explanations in the Discussion section (from page 19, line 352, to page 20, line 362). A major revision was to specify that the population of this study was women who had undergone the self-sampling HPV test, rather than the general population. Additionally, the Abstract (page 4, lines 49–50), the Statistical analysis section (page 9, lines 160–162), the Results section (from page 10, lines 195–197), and the Conclusion section (page 20, line 374) were also revised.

# Reviewer 1

# Comment 1

I have a question about patient selection criteria. Please explain briefly why you excluded patients who received regular cervical cancer screening and included only those who had not been screened for three years or more. We believe that asking patients who are regularly screened for their opinion on self-sampling is also helpful and has the advantage of reducing selection bias.

# Response 1

Thank you for your comment. Self-sampling HPV testing has already been implemented in other countries, such as the Netherlands and Denmark, and the test has only been recommended for regular screening non-responders. Therefore, we assumed that a self-sampling HPV test, if implemented in Japan, would be performed by regular screening non-responders. We added this explanation from page 6, lines 110–112.

# Comment 2

It is important to note that more than 60% of patients had negative feelings about self-sampling in the results of this study. The authors simply state that “This may reflect the Japanese personality (Page 18, line 302)” as one of the reasons. This sentence alone does not have enough grounds for the reason. It would be desirable to include suggestions on improving this result, which I believe will give readers more valuable tips.

# Response 2

We agree with you and have deleted this sentence due to the lack of grounds for the reason. As already described in the text, to eliminate women’s concerns related to the accuracy of the self-sampling HPV test, it is necessary to provide information related to the self-sampling test in advance, such as the probability of invalid test results and the accuracy of the tests. We recently reported that the probability of invalid test results was rare among Japanese women; accordingly, we cited this article in the text (page 17, lines 303–305).

# Comment 3

The statement by the authors, "There would be women with negative impressions of the self-sampling HPV test in the general population, such women might also change their minds of experience.'' (Page 21, lines 359-361) I think this is an essential suggestion for the spread of self-sampling in Japan in the future. I think it would be more interesting for the readers if you mentioned it as a Finding instead of a Limitation and put forward proactive proposals for raising awareness of patients in Japan in the Discussion.

# Response 3

Thank you for your comment. We are glad that you recognize the importance of this study. In line with your comment, the descriptions were indicated in the Discussion section as a finding after the explanation of this study’s strengths. Additionally, a suggestion to promote the self-sampling test in Japan was added (from page 20, lines 364–372).

# Comment 4

Overall, the text is large and redundant, so please try to keep it concise by omitting content that is not directly related to this research as much as possible. For example, it is not common for research papers targeting populations to develop an argument based on individual responses to the questionnaire (Page13, line219-223Pape21, line357-359). We also agree that the description about COVID-19 (page 21, line 367-372) is essential, but it is not directly related to the results of this study, so I recommend that you delete it.

# Response 4

Thank you for your comment. We deleted the descriptions based on individual responses in the Results section and those related to COVID-19 in the Discussion section. Additionally, some sentences in the Discussion section were deleted.

# Reviewer 2

# Comment 1

The proportion of those who chose the self-sampling HPV test was very low (16.3%), which may cause sampling bias. Since the main purpose of this manuscript is to evaluate the positive impression of the self-sampling HPV test, this bias is a major problem that directly affects the conclusion of the manuscript, and is also insufficiently addressed in the limitation.

The table 1 describes only the background of the intervention group. It is possible that the intervention and non-intervention groups have different backgrounds.

# Response 1

Thank you for your comment. We also recognize the selection bias in this study. In line with your comment, we revised Table 1 (from page 10, line 199, to page 11, line 204) and added explanations in the Discussion section (from page 19, line 352, to page 20, line 362). A major revision was to specify that the population of this study was women who had undergone the self-sampling HPV test, rather than the general population. Additionally, the Abstract (page 4, lines 49–50), the Statistical analysis section (page 9, lines 160–162), the Results section (from page 10, lines 195–197), and the Conclusion section (page 20, line 374) were also revised.

# Comment 2

Ⅼ225-227 states that older people tend to experience more difficulty regarding the pain of self-sampling HPV tests, while Ⅼ334-339 emphasizes that there is no association with age in the collection of HPV tests. The logic is inconsistent.

# Response 2

Thank you for your comment. We agree with you.

We would like to advocate that self-sampling HPV testing is an acceptable method regardless of age and duration without screening. Therefore, we deleted statements explaining that self-sampling HPV testing is more acceptable to the younger generation and revised the explanation in the Discussion section (page 19, lines 335–341).

# Comment 3

Table 2 and other tables have many items but are redundant and need to be made more compact. It would be desirable to consider changing to bar graphs, etc., which are visually easy to understand.

# Response 3

According to your comment, we changed Table 2 to a graph (from page 12, lines214–215). The other tables remain unchanged because graphs would not allow for the presentation of some meaningful information.
