# Supplementary material for: Acceptability of self-sampling human papillomavirus test for cervical cancer screening in Japan: A questionnaire survey in the ACCESS trial
Source: PLoS One. 2023 Jun 8;18(6):e0286909. doi: 10.1371/journal.pone.0286909 (PMC10249862; doi:10.1371/journal.pone.0286909)
Supplement: Attachment — Submitted filename: Response_to_Reviewers_20230522.docx [file pone.0286909.s005.docx]

# Responses to the Editor’s Comments

Submission ID: PONE-D-22-28147

Title: Acceptability of self-sampling human papillomavirus test for cervical cancer screening in Japan: A questionnaire survey in the ACCESS trial

We appreciate the Editor’s comments toward improving our paper. We have revised the manuscript to address all the comments, and our point-by-point responses are provided below. We have included the **page and line numbers of the revisions made in the main text**. Revisions in response to the Editor’s comments are **highlighted in yellow** in the revised manuscript with tracked changes.

# Point-by-point responses

# Academic editor

# Comment 1

Page 6, Line 107: It is unclear to define an inclusion criterion by stating “women considered the target population for cervical cancer screening by Ichihara City in 2021’. Please provide details on what this criterion really entails.

# Response 1

Thank you for your comment. The target population for cervical cancer screening provided by Ichihara City in 2021 was women whose age was an even number. The city set this eligibility requirement to offer cervical cancer screening once every two years to all women. We have added the relevant explanation in the manuscript (page 6, lines 111–114).

# Comment 2

Page 8, Line 156: The numbers of participants falling into the category of ‘without registration’ are substantial as shown in different tables (Tables 1&2&3). The unknown composition of this category has the potential to bias the results. Could the authors provide information on the possible reasons that led to women not being registered and discuss how the study results would be affected under different scenarios?

# Response 2

Thank you for your comment. As our descriptions were misleading, we have revised them and the changed the category’s name (page 8 lines 161–162). The category “no screening records” comprised women who had no records regarding cervical cancer screening provided by Ichihara City, such as the date of the screening. As described in the Discussion section (page 17, lines 294–300), Japan does not have a registry system for cervical cancer screening. Therefore, we could not truly determine screening non-responders and under-responders. Therefore, there is a possibility that all the categories pertaining to the duration without screening included women who had undergone the screening provided by other site, not the city. Explanations of the sensitivity analyses regarding this point are presented in the Statistical analysis (page 9, lines 174–177), Results (page 14, lines 263–265), and Discussion (from page 18, line 328–page 19, line 336) sections.

# Comment 3

Page 9, Statistical Analysis: Please add a description of how the trend test analysis was applied in the study.

# Response 3

We have added the explanation in the Statistical analysis section (page 9, lines 179–181).

# Comment 4

Page 10, Line 195-197: There appears to be a lack of sufficient description and interpretation about the data presented in Table 1. For instance, although there is a statistically significant difference in the mean ages between the groups in Table 1, a difference of 44.1 vs. 44.7 may not be clinically meaningful. What are the implications of the differences in the distributions of age and duration without screening?

# Response 4

We have described these differences in the Discussion section (page 20, lines 363–365). As you have mentioned, these differences may be trivial. However, because of the significant differences, we accepted the possibility that the sample included this study did not represent general population. This has been explained in the manuscript (page 20, lines 359–369).

# Comment 5

Page 12, Line 232-233: There are no 49.1% vs. 89.3% shown in Table 2. Should that be 46.9% vs. 86.7%?

# Response 5

Thank you for bringing this to our attention. Per your comment, we have corrected these numbers (page 12, line 238–page 13, line 239).

# Comment 6

Page 18, Line 315: Please rewrite this sentence ‘the knowledge was less in vulnerable women with lower screening rates’. Do you mean that women who had lower screening rates and are considered more vulnerable had less knowledge about HPV?

# Response 6

Thank you for your comment. We have revised the description as presented in page 18, lines 321–324. Additionally, we have cited more articles (reference numbers 38 and 39) in the revised manuscript.
